# Assessment of Color Stability and Translucency of Five High-Translucent Resin Composites

**DOI:** 10.4317/jced.64173

**Published:** 2026-07-29

**Authors:** José Giancarlo Tozo-Burgos, Marco Sánchez-Tito

**Affiliations:** 1 Research Group on Dental Biomaterials and Natural Products, Faculty of Health Sciences, Universidad Privada de Tacna, Tacna, Peru

## Abstract

**Background:**

To evaluate and compare the color stability and translucency of high-translucent resin composites after exposure to distilled water and coffee.

**Materials and Methods:**

Standardized disc-shaped specimens of five commercially available translucent resin composites (n = 60; 12 specimens per group) were fabricated and evaluated at three time points: baseline (T0), after 30 days in distilled water (T1), and after an additional 30 days in coffee (T2). Color was measured using a spectrophotometer and expressed as E00, while translucency was assessed using the translucency parameter (TP). Data were analyzed using non-parametric tests. The level of significance was set at 5%.

**Results:**

All materials showed a significant increase in color change after coffee exposure (p < 0.05). Beautifil II exhibited the highest discoloration at T2-T0 (17.76 [2.18]), followed by Forma Trans (14.93 [3.70]), whereas Opallis T Neutral showed the lowest values (8.31 [2.84]). At T1-T0, Opallis T Neutral also presented the lowest color change (1.42 [0.67]). Regarding translucency, Filtek Z350 XT Trans showed the highest TP values at T2 (15.38 [2.74]), while several materials, particularly Beautifil II (-4.08 [4.35]) and PALFIQUE LX5 (-4.60 [2.96]), exhibited reductions in TP after coffee immersion. Opallis T Neutral showed minimal variation in translucency (-0.19 [1.19]).

**Conclusions:**

High-translucent resin composites showed significant differences in optical stability. Coffee exposure increased discoloration and reduced translucency in most materials. Opallis T Neutral demonstrated the best overall performance.

## Introduction

Resin composites are extensively utilized in contemporary dentistry, primarily due to their esthetic qualities, favorable physico-mechanical properties, and cost-effectiveness (1 , 2). Previous studies have demonstrated that extrinsic factors, including the consumption of pigmented beverages such as coffee, tea, and wine, can significantly affect the optical properties of resin composites (3 - 7). Color changes in resin composites have been associated with intrinsic factors related to their composition, such as the type of monomer in the organic matrix, the percentage and size of filler particles, the photoinitiator system, and the degree of conversion following polymerization. These factors directly influence the material's optical properties. Additionally, matrices containing more hydrophilic monomers, such as Bis-GMA or TEGDMA, exhibit increased water sorption, which promotes intrinsic staining and accelerates esthetic degradation (8 - 12). Conversely, studies simulating intraoral thermal changes have shown that these variations contribute to the optical and structural degradation of restorative materials (13 , 14). These conditions can induce microcracks, which increase the absorption of pigmented liquids and promote the progressive loss of essential optical properties, such as translucency (13 - 15). Such factors may compromise the esthetic and functional longevity of restorations over the medium and long term, prompting the ongoing development of new restorative materials with formulations designed to optimize both optical and physico-mechanical properties (16). Despite these advancements, specific groups of resin composites, particularly those with a translucent appearance, have received limited investigation. These materials are primarily utilized for the reconstruction of incisal edges in anterior teeth (17 , 18). However, there is insufficient evidence regarding their characteristics and optical behavior when exposed to extrinsic factors such as pigmented beverages. In this context, the study by Piccoli et al. stands out as one of the most comprehensive investigations to date, evaluating the color and translucency of different resin composites, including Filtek Z350 XT, Opallis, Amelogen Plus, and IPS Empress Direct, after storage in water and coffee. The results showed that IPS Empress Direct exhibited the highest susceptibility to optical changes, followed by Amelogen Plus, with both materials presenting significant alterations in color and translucency after coffee exposure (19). Additionally, another study reported that translucent resin composites exhibit inferior physico-mechanical properties compared to universal composites, showing behavior similar to flowable composites, mainly due to their lower inorganic filler content (20). However, currently available evidence evaluating high-translucent resin composites remains limited, particularly regarding the simultaneous assessment of color stability, translucency behavior, and CIELAB coordinate changes after sequential exposure to artificial saliva and coffee. Furthermore, recently introduced translucent composites with different resin matrices and filler technologies have not been extensively compared under standardized aging protocols. Given the limited available evidence, the present study aimed to evaluate and compare the color stability and translucency of various translucent resin composites following exposure to distilled water and coffee.

## Materials and Methods

-Study Design, Ethical Considerations, and Sample Size This in vitro experimental study was approved by the Ethics Committee of the Faculty of Health Sciences at the Universidad Privada de Tacna (FACSA-CEI/034-04-2025). Ten experimental groups were established to assess the influence of storage conditions (30 days in distilled water and 30 days in coffee) on color difference (E00) and translucency parameter (TP) in five commercially available translucent resin composites. The materials evaluated are listed in Table 1.

Table 1

Sample size estimation was performed using G*Power software version 3.1.3 (Heinrich Heine University, Düsseldorf, Germany), based on translucency values reported in a previous study (19). A pooled standard deviation was used to calculate an effect size (f) of 0.49. Assuming a significance level () of 0.05, a statistical power (1) of 0.90, and a repeated measures ANOVA design, the initial required sample size was estimated at 50 specimens. To ensure balanced allocation across the five experimental groups and to enhance the robustness of the analysis, the sample size was increased to 60 specimens (n = 12 specimens per resin composite group). This adjustment resulted in an increased statistical power of approximately 0.96, thereby improving the sensitivity of the study to detect significant differences among groups. -Specimen Preparation Twelve disc-shaped specimens per group were fabricated using a metallic mold with an internal diameter of 8 mm and a thickness of 2 mm (17). The resin was inserted into the mold in a single increment, covered with a Mylar strip, and gently pressed with a glass slide to achieve a flat surface. The specimens were light-cured for 20 seconds (21) using a VALOTM Cordless curing unit (Ultradent Products Inc., South Jordan, UT, USA) at an irradiance of 1200 mW/cm². The specimens were then stored in distilled water for 24 hours at room temperature. Specimens presenting visible defects, including air bubbles, marginal irregularities, surface cracks, polishing defects, or thickness variation greater than ±0.1 mm, were excluded and replaced prior to optical analysis. Subsequently, the specimens were polished on both surfaces using the Super-Snap system (Shofu, Kyoto, Japan), following a sequential progression of color-coded abrasive discs: black (coarse), violet (medium), green (fine), and red (superfine). Each polishing step was performed under light pressure with smooth, continuous movements for approximately 30 seconds per disc. The final thickness of each specimen was verified using a digital micrometer (resolution: 0.001 mm) (Digital Micrometer IP65, Asimeto®, Mooresville, NC, USA). The specimens were then stored in distilled water until use (22). -Color Stability Color measurements were conducted using the CIELAB color system and a VITA Easyshade spectrophotometer (VITA Zahnfabrik, Bad Säckingen, Germany). Specimens were placed on a standard background (L* = 52.2, a* = 0.4, b* = -4.6) supplied by the manufacturer. Each measurement was performed in triplicate by positioning the spectrophotometer tip at the center of each specimen (19), under standard illuminant D65 (CIE). Color was evaluated at three time points. Baseline values (T0 = control) were recorded 24 hours after polymerization, following gentle air-drying of specimens with oil-free air. Subsequently, each specimen was immersed in 5 mL of distilled water at 37 °C for 30 days, after which a second measurement (T1) was performed. A third measurement (T2) was obtained after immersion in a coffee solution at 37°C for an additional 30 days. The coffee solution (Nescafé Tradición, Nescafé®, Nestlé Brasil Ltda., Araras, SP, Brazil) was prepared by dissolving 4 g of coffee in 300 mL of boiling distilled water with constant stirring. After cooling to room temperature, the solution was filtered using No. 1 filter paper (Whatman®, Cytiva, Marlborough, MA, USA) before use (23). The immersion medium was renewed every 24 hours. Color differences after exposure were calculated using the CIEDE2000 formula (24 , 25):

$${\Delta E}_{00}={\left[{\left(\frac{\Delta L}{K_{L}\,S_{L}}\right)}^{2}+{\left(\frac{\Delta C}{K_{C}\,S_{C}}\right)}^{2}+{\left(\frac{\Delta H}{K_{H}\,S_{H}}\right)}^{2}+R_{T}\,\left(\frac{\Delta C}{K_{C}\,S_{C}}\right)\,\left(\frac{\Delta H}{K_{H}\,S_{H}}\right)\right]}^{\frac{1}{2}}$$

Where L, C, and H denote the differences in lightness, chroma, and hue between two measurements. The rotation term (RT) accounts for the interaction between chroma and hue differences in the blue region, thereby enhancing the accuracy of color difference estimation. The terms KLSL, KCSC, and KHSH are weighting functions that adjust the differences in each coordinate within the CIEDE2000 model. The parameters KL, KC, and KH were set to 1, consistent with previous reports (26). -Translucency Stability The translucency parameter (TP) of each specimen was calculated using the following formula (27):

$$\mathrm{TP}={\left[{\left({L*}_{w}-{L*}_{b}\right)}^{2}+{\left({a*}_{w}-{a*}_{b}\right)}^{2}+{\left({b*}_{w}-{b*}_{b}\right)}^{2}\right]}^{\frac{1}{2}}$$

The variable w represented the CIELAB values measured over a white background (L* = 93.7, a* = 1.2, b* = 0.8), while b corresponded to values measured over a black background (L* = 8.6, a* = -0.7, b* = -1.0). The translucency parameter (TP) was calculated for each storage period. To maintain optical continuity between the translucent resin and the opaque background, a water-based glycerin gel was placed between the specimen and the background (17). -Statistical Analysis Statistical analyses were conducted using GraphPad Prism software (GraphPad Software, San Diego, CA, USA). Data distribution was evaluated with Q-Q plots and the Shapiro-Wilk test. Due to deviations from normality, non-parametric tests were utilized. Data are presented as median and interquartile range (IQR). For color change (E00), comparisons among materials at each time interval (T1-T0 and T2-T0) were made using the Kruskal-Wallis test, followed by Dunn's post hoc test. Intra-material comparisons between intervals (T1-T0 vs. T2-T0) were performed using the Wilcoxon signed-rank test. For the translucency parameter (TP), comparisons among materials at each time point (T0, T1, and T2), as well as for the differences (TP: T1-T0 and T2-T0), were conducted using the Kruskal-Wallis test followed by Dunn's test. Intra-material comparisons across time points (T0, T1, and T2) were assessed using the Friedman test, followed by Dunn's multiple comparisons test. The significance level was set at 0.05.

## Results

Table 2 shows the color differences (E00) following aging. At T1-T0, significant differences were identified among the materials (p < 0.05), with Forma Trans and PALFIQUE LX5 displaying the highest values and Opallis T Neutral the lowest.

Table 2

**Figure 1:**
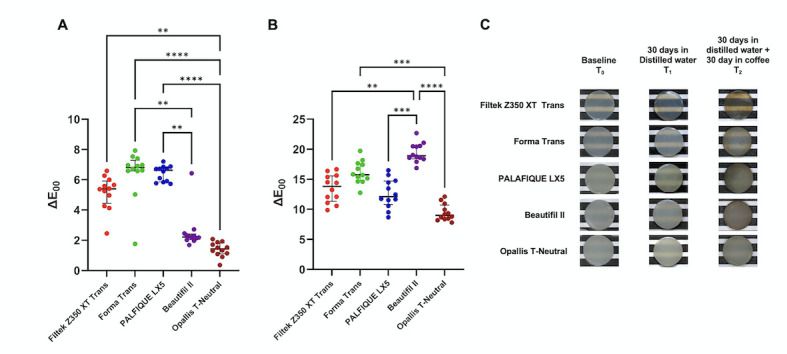
ΔE00 values of high translucent resins after 30 days in distilled water (A) and after additional 30 days in coffee (B). (C) Representative images of each material at baseline (T0), after distilled water immersion (T1), and following coffee exposure (T2).

At T2-T0, all materials showed increased E00 (p < 0.05); Beautifil II and Forma Trans exhibited the highest values, while Opallis T Neutral remained the lowest (8.31 [2.84]). For all materials, E00 was significantly higher at T2-T0 compared to T1-T0 (p < 0.05). Figure 1 illustrates these results, showing increased E00 values after coffee immersion (Fig. 1B) relative to distilled water storage (Fig. 1A), along with corresponding visual changes in the representative specimens (Fig. 1C).

1 Screenshot

**Figure 2:**
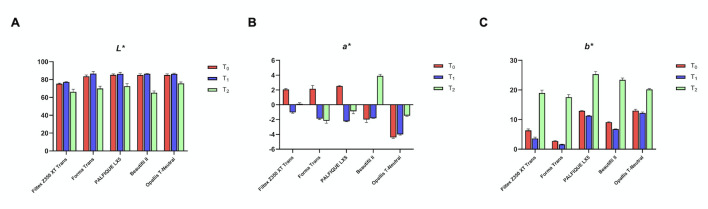
CIE L*, a*, and b* values of high-translucent resin composites at T0, T1, and T2.

Analysis of color coordinates indicated a reduction in L* values following aging, especially in Forma Trans and PALFIQUE LX5 (Fig. 2A).

2

Most materials demonstrated a shift of the a* parameter toward negative values over time; however, Opallis T Neutral exhibited an increase toward positive values at T2 (Fig. 2B). All materials showed an increase in b* values, with PALFIQUE LX5 and Beautifil II displaying the highest values (Fig. 2C). Table 3 shows the translucency parameter (TP) values at each evaluated time point. At baseline (T0), Forma Trans demonstrated the highest TP values (p < 0.05).

Table 3

**Figure 3:**
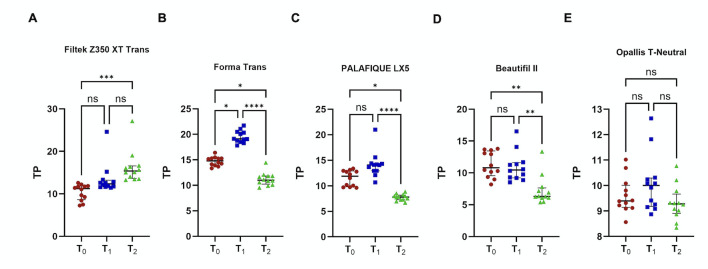
Translucency parameter (TP) variation of resin composites across storage times.

Following distilled water storage (T1), both Forma Trans and Filtek Z350 XT Trans showed higher TP values than the other materials (p < 0.05). At T2, Filtek Z350 XT Trans maintained the highest TP values, while Opallis T Neutral showed the lowest. For TP, Forma Trans and Filtek Z350 XT Trans showed an increase in TP at T1-T0 (p < 0.05). In contrast, at T2-T0, several materials, particularly Forma Trans, PALFIQUE LX5, and Beautifil II, exhibited reduced TP values after coffee immersion. Figure 3 depicts the changes in the translucency parameter (TP) over time for each resin composite.

3

For each material, TP values at T0, T1, and T2 were compared using the Friedman test, followed by Dunn's multiple comparisons test. Significant temporal differences were identified in most materials, except for Opallis T Neutral, which did not exhibit significant changes. Notably, Filtek Z350 XT Trans demonstrated a progressive increase in TP values over time, whereas Opallis T Neutral exhibited minimal variation throughout the aging protocol.

## Discussion

This study assessed the optical stability of five high-translucent resin composites following sequential storage in distilled water and coffee. All materials demonstrated significant color changes over time, with a pronounced increase after coffee immersion. In contrast, translucency exhibited material-dependent variation; some resins showed an initial increase after distilled water storage, followed by a decrease after coffee exposure, while others remained relatively stable. In terms of color stability, all materials exhibited significantly higher E00 values at T2-T0 compared to T1-T0, confirming the pronounced staining effect of coffee. These results align with previous studies that identify coffee as a highly aggressive staining agent due to its capacity to promote both surface adsorption and subsurface absorption of chromogenic compounds (3 - 7 , 10). Notable differences among materials were observed: Beautifil II and Forma Trans displayed the highest E00 values after coffee immersion, while Opallis T Neutral consistently exhibited the lowest values. Piccoli et al. (19) similarly reported that highly translucent composites vary significantly in their susceptibility to staining after exposure to pigmented media. More recently, Almohareb et al. (28) demonstrated that artificial staining procedures, particularly coffee immersion, significantly compromise both color stability and translucency in resin-based composites. Similarly, Karaman et al. (29) observed clinically perceptible discoloration after coffee exposure in highly esthetic resin composites. These findings are consistent with the present study, in which all evaluated materials exhibited substantial increases in E00 values after coffee aging. Variations in material composition and microstructure account for these differences. High-translucent composites are generally formulated with reduced pigment concentration and optimized filler systems to improve light transmission. However, these modifications may also affect their interaction with staining agents (17 , 30). The composition of the organic matrix is critical, as monomers such as Bis-GMA and TEGDMA exhibit greater hydrophilicity and water sorption, which facilitate pigment diffusion and internal discoloration (8 - 12). In contrast, systems that incorporate more hydrophobic monomers or alternative dimethacrylates may exhibit reduced susceptibility to staining (16). Furthermore, filler content, particle size, and distribution influence surface roughness and light-scattering, thereby affecting stain retention and optical stability (5 , 10 , 17). Materials with lower filler loading or weaker filler-matrix coupling are more susceptible to pigment uptake and optical degradation (20). These factors likely contributed to the greater color stability observed in Opallis T Neutral and the increased susceptibility of Beautifil II and Forma Trans in the present study. From a clinical perspective, the magnitude of the observed color change is highly relevant. In most cases, E00 values surpassed widely recognized perceptibility and acceptability thresholds, particularly following coffee immersion (31 , 32). These findings indicate that, although high-translucent composites exhibit favorable esthetic properties initially, they are susceptible to clinically perceptible discoloration over time when exposed to common dietary staining agents. Analysis of CIE color coordinates corroborates these results. A reduction in L* values in several materials demonstrates a general darkening, while an increase in b* values indicates a shift toward yellow tones, consistent with the chromatic characteristics of coffee. Variations in a* were material-dependent, reflecting differences in hue behavior among composites. Collectively, these results confirm that discoloration is a multidimensional process involving concurrent changes in lightness, chroma, and hue, as previously reported (5 , 26). The translucency results revealed a complex pattern. Some materials, including Filtek Z350 XT Trans and Forma Trans, demonstrated increased translucency parameter (TP) following distilled water storage. In contrast, several materials, notably Forma Trans, PALFIQUE LX5, and Beautifil II, exhibited reduced translucency after immersion in coffee. These observations align with previous studies indicating that aging and immersion media can differentially influence translucency based on material composition (14 , 15 , 22). The observed increase in translucency after distilled water storage may be attributed to post-curing effects or temporary changes in the refractive index resulting from water diffusion into the resin matrix (22 , 33). The reduction in TP observed in Beautifil II and PALFIQUE LX5 after coffee exposure also agrees with the findings of Almohareb et al. (28) and Karaman et al. (29), who reported that prolonged contact with pigmented beverages may compromise translucency stability due to pigment absorption and alterations in light transmission behavior. In contrast, the reduction in translucency following coffee exposure may result from increased light scattering due to pigment uptake, water sorption, and microstructural changes within the composite (7). Translucency is determined by the refractive index compatibility between the organic matrix and filler particles; any disruption of this balance leads to optical changes (14 , 22). Interestingly, Filtek Z350 XT Trans demonstrated a progressive increase in TP throughout the aging protocol, suggesting that the optical behavior of highly translucent composites may vary considerably according to filler technology and resin matrix composition. These findings are clinically significant, as even minor variations in translucency can be perceptible and influence the esthetic integration of restorations (15 , 34). This consideration is especially relevant for high-translucent composites, which are frequently utilized in anterior restorations and incisal edge reconstruction, where achieving optical harmony with adjacent enamel is essential (17 , 18). Consequently, the long-term stability of both color and translucency should be prioritized when selecting restorative materials. Clinicians should therefore consider not only the initial esthetic appearance of translucent composites, but also their long-term susceptibility to staining and translucency alteration when treating patients with frequent consumption of pigmented beverages such as coffee. This study is subject to limitations associated with its in vitro design. Using only one staining solution and excluding additional aging factors, such as thermocycling or mechanical wear, limits the direct applicability of the findings to clinical scenarios. Moreover, oral environmental conditions such as pH fluctuation, salivary enzymes, biofilm accumulation, and toothbrushing abrasion were not simulated and may influence the long-term optical behavior of restorative materials. Nevertheless, the standardized methodology and the combined assessment of color stability, translucency parameter, and CIELAB coordinates yield clinically relevant insights into the performance of high-translucent composites. As a conclusion of this study, high-translucent resin composites exhibited significant differences in their optical stability following aging. Coffee exposure resulted in a marked increase in color change across all materials and a reduction in translucency in most cases, although the magnitude of these effects was material-dependent. Among the evaluated materials, Opallis T Neutral demonstrated the best overall performance, showing the lowest color change values and minimal alterations in translucency. These findings highlight the importance of considering not only the initial esthetic properties but also the long-term optical stability when selecting restorative materials.

## Figures and Tables

**Table 1:** Characteristics of the high-translucent resin composites included in this study.

Resin Composite	Origin	Characteristics
Filtek™ Z350 XT Translucent CT (3M)	United States (St. Paul, Minnesota)	Resin matrix composed of Bis-GMA (bisphenol A-glycidyl methacrylate), UDMA (urethane dimethacrylate), TEGDMA (triethylene glycol dimethacrylate), and Bis-EMA (ethoxylated bisphenol A dimethacrylate). Contains 20 nm silica nanoparticles and silica/zirconia nanoclusters (0.4–0.6 µm). Inorganic filler loading: 78.5 wt%.
Forma Trans (Ultradent)	United States (South Jordan, Utah)	Nano-hybrid composite formulated with an organic matrix based on Bis-GMA, UDMA, TEGDMA, and Bis-EMA, and inorganic fillers of zirconia and ytterbium trifluoride with particle sizes ranging from 0.014 to 3 µm; filler content approx. 72–75 wt% and 25–28 wt% organic matrix.
PALFIQUE LX5 CE (Tokuyama)	Japan (Tokyo)	Resin composite with 82 wt% (71 vol%) filler composed of silica-zirconia and composite filler. Incorporates submicron spherical particles with an average size of 0.2 µm.
Beautifil II (Shofu)	Japan (Kyoto)	Bioactive nano-hybrid composite incorporating S-PRG (Surface Pre-Reacted Glass-ionomer) technology, allowing fluoride release and recharge. Contains discrete nano-fillers (10–20 nm), with a filler loading of 83.3 wt%.
Opallis T-Neutral (FGM)	Brazil (Joinville, Santa Catarina)	Nano-hybrid composite formulated with Schott glass particles and silica nanoparticles with an average size of 0.5 µm.

1

**Table 2:** Color Differences (ΔE00) of high translucent resin composites after two aging intervals.

Resin	ΔE00	
	T1 – T0	T2 – T0
Filtek Z350 XT Trans	5.39 [1.21]aA	11.40 [3.13]bcB
Forma Trans	6.80 [0.60]aA	14.93 [3.70]abB
PALFIQUE LX5	6.62 [0.92]abA	11.65 [2.95]bcB
Beautifil II	2.21 [0.30]bcA	17.76 [2.18]aB
Opallis T Neutral	1.42 [0.67]cA	8.31 [2.84]cB

Values are expressed as median [interquartile range (IQR)]. Different lowercase letters within the same column indicate statistically significant differences among materials, according to the Kruskal–Wallis test followed by Dunn’s post hoc test (p < 0.05). Different uppercase letters within the same row indicate statistically significant differences between time intervals, according to the Wilcoxon signed-rank test (p < 0.05).

**Table 3:** Translucency parameter (TP) expressed as medians [IQR] and ΔTP values at Different ageing stages in high translucent resin composites.

Resin	Aging condition			ΔTP	
	T0	T1	T2	T1 – T0	T2 – T0
Filtek Z350 XT Trans	11.24 [2.95]b	12.36 [1.41]b	15.38 [2.74]a	3.07 [3.78]b	4.89 [3.89]a
Forma Trans	14.81 [1.27]a	19.10 [1.75]a	11.01 [1.47]b	4.22 [3.05]a	-3.44 [1.02]b
PALFIQUE LX5	11.88 [2.96]c	14.07 [1.40]b	7.80 [0.83]c	1.37 [3.69]b	-4.60 [2.96]b
Beautifil II	10.81 [3.56]c	10.45 [2.27]c	6.31 [1.37]c	-0.34 [4.04]c	-4.08 [4.35]b
Opallis T-Neutral	9.39 [0.76]b	9.99 [1.07]c	9.27 [0.60]d	0.38 [1.91]c	-0.19 [1.19]c

Different superscript letters indicate statistically significant differences between groups (p < 0.05), according to Dunn’s post hoc test following the Kruskal–Wallis test.
